# A Cross-Sectional Study on Relationships Between Depression and Anxiety in Hidradenitis Suppurativa Patients and Disease Severity, Subjective Symptoms and Quality of Life

**DOI:** 10.3390/jcm15020700

**Published:** 2026-01-15

**Authors:** Marta Szepietowska, Piotr K. Krajewski, Przemyslaw Pacan, Anna Wojas-Pelc, Lukasz Matusiak, Andrzej K. Jaworek

**Affiliations:** 1 Department of Dermatology, Jagiellonian University Medical College, 31-503 Cracow, Poland; anna.wojas-pelc@uj.edu.pl; 2 Doctoral School of Medical and Health Sciences, Jagiellonian University Medical College, 31-503 Cracow, Poland; 3 Division of Dermatology, Venereology and Clinical Immunology, Faculty of Medicine, Wroclaw University of Science and Technology, 50-370 Wroclaw, Poland; piotr.krajewski@pwr.edu.pl (P.K.K.); lukasz.matusiak@pwr.edu.pl (L.M.); 4 Department of Dermato-Venereology, 4th Military Hospital, 50-981 Wroclaw, Poland; 5 Lübeck Institute of Experimental Dermatology, University of Lübeck, 23562 Lübeck, Germany; 6 Department of Psychiatry, Institute of Medical Sciences, Rzeszow University, 35-310 Rzeszow, Poland; ppacan@ur.edu.pl

**Keywords:** hidradenitis suppurativa, comorbidities, depression, anxiety, quality of life

## Abstract

**Background/Objectives**: Hidradenitis suppurativa (HS) is a chronic, inflammatory, and recurrent disorder of the pilosebaceous unit with numerous comorbidities. Growing evidence suggests that depression and anxiety occur more frequently in HS patients, yet their relationship with clinical severity and especially subjective symptoms remains insufficiently understood. The aim of this study was to assess the prevalence and severity of probable depressive and anxiety symptoms in Polish patients with HS and to examine their associations with clinical disease severity, pain and itch intensity, and quality of life (QoL). **Methods**: Eighty-four HS patients were included in this cross-sectional study. Disease severity was assessed using Hurley staging and the IHS4. Pain and itch intensity were evaluated using the Numeric Rating Scale (NRS). Psychological assessment included self-administered screening questionnaires, such as PHQ-9 and HADS-D for depression and GAD-7 and HADS-A for anxiety. QoL was measured using DLQI and HiSQOL instruments. Statistical analyses were performed with *p* < 0.05 considered significant. **Results**: Possible depressive disorders were identified in 25.0% of patients. PHQ-9 and HADS-D scores differed significantly across Hurley stages and correlated positively with IHS4. Possible anxiety disorder according to GAD-7 criteria was present in 15.5% of patients. Both GAD-7 and HADS-A correlated with IHS4. They also showed correlations with pain and/or itch intensity. All psychological measures showed strong correlations with both QoL instruments. **Conclusions**: Depression and anxiety seem to be common in HS and closely associated with clinical severity and reduced QoL. Their relation with pain and itch requires further studies. These findings underscore the need for multidisciplinary management in HS care.

## 1. Introduction

Hidradenitis suppurativa (HS) is a chronic, inflammatory, and recurrent dermatosis of the pilosebaceous unit that predominantly affects apocrine gland-bearing areas such as the axillae, groin, gluteal region, and perineum. The disease is characterized by painful, deep-seated nodules, abscesses, tunnels, and disfiguring scarring, frequently accompanied by pain and pruritus. Its onset typically occurs in young adulthood, with the highest prevalence between puberty and 40 years of age [1,2]. Although the estimated prevalence of HS in Europe is around 1% [3,4], global epidemiological data remain inconsistent, partly due to misclassification, delayed diagnosis, and limited disease recognition among healthcare providers. Patients often experience diagnostic delays of several years and numerous consultations across specialties before receiving appropriate care [5,6,7].

The pathogenesis of HS is multifactorial, involving genetic predispositions, environmental influences, and dysregulated immune pathways. Increasing evidence points to a central role of pro-inflammatory cytokines and a potential autoimmune component [1]. Beyond the cutaneous manifestations, HS is associated with a broad spectrum of systemic comorbidities, including obesity, metabolic syndrome, cardiovascular disease, inflammatory bowel disease, and spondyloarthropathies [1,8]. The physical burden of chronic inflammation, restricted mobility, and lesions in intimate body regions significantly impairs daily functioning, social participation, and sexual health [9,10,11,12].

Importantly, the burden of HS extends well beyond physical symptoms. Individuals with HS experience profound psychosocial morbidity, with substantial negative effects on quality of life (QoL), employment, interpersonal relationships, and overall well-being [9,12].

Numerous studies indicate that psychiatric disorders, particularly depression, anxiety, substance use disorders, and suicidality, occur disproportionately in this population [13,14]. Previous studies have demonstrated that over 20% of HS patients screen positive for depressive and anxiety symptoms [13,14]. Importantly, these psychiatric conditions contribute substantially to the overall disease burden and are closely linked to profound impairment in QoL, which in HS is among the most severely affected compared to other dermatological conditions. The study conducted by our group clearly showed that HS patients experienced a very large effect on their QoL, with 22% of HS subjects reporting an extremely large effect [12]. Beyond the mere presence of psychiatric symptoms, increasing attention has been paid to the complex interplay between psychological distress and hallmark HS symptoms, including pain. Pain, in particular, is one of the most disabling symptoms of HS and has been identified as a key independent predictor of reduced QoL, not only in dermatological diseases but also in patients with depression, where it significantly influences both baseline QoL and its changes over time. Recent evidence indicates that pain severity may predict deterioration in QoL independently of depressive symptom severity, while simultaneously amplifying anxiety and depressive symptoms, thereby creating a self-perpetuating cycle of physical and psychological distress [15].

Despite growing recognition of these relationships, data simultaneously addressing depression, anxiety, objective disease severity, subjective symptoms such as pain and itch, and QoL within a single HS cohort remain scarce. Therefore, a more comprehensive understanding of how these domains interact is needed to introduce holistic and multidisciplinary approaches to HS management. The present study aims to address this gap by evaluating the prevalence and severity of depressive and anxiety symptoms in patients with HS and by exploring their associations with clinical disease severity, pain and itch intensity, and QoL.

## 2. Materials and Methods

### 2.1. Study Design, Settings, Patients’ Characteristics and Ethics

**Figure 1:**
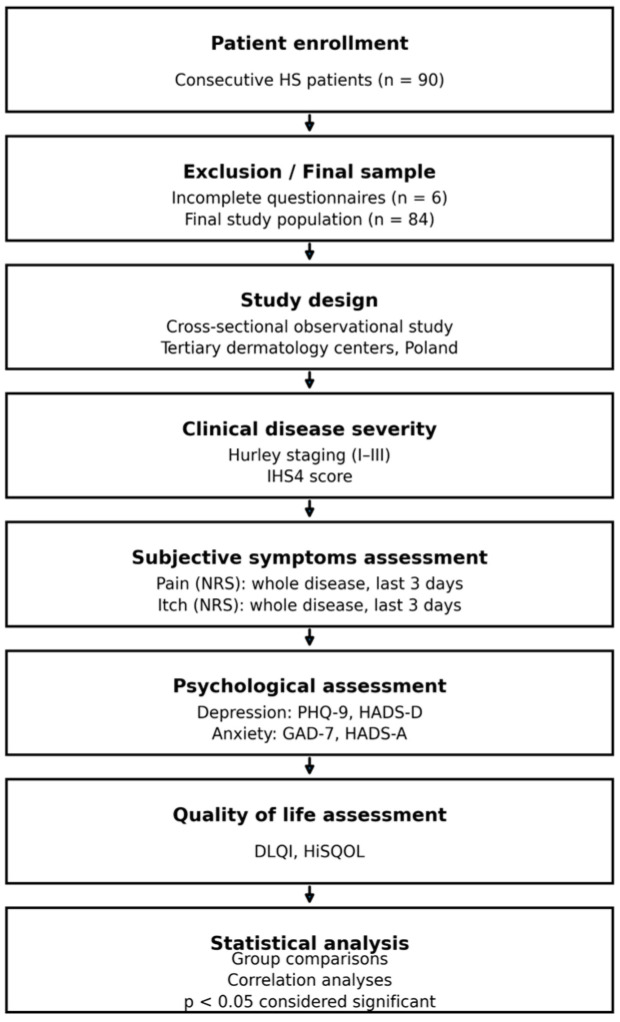
Study design. Consecutive patients with HS were enrolled and assessed for clinical disease severity, subjective symptoms (pain and itch), psychological status (depression and anxiety), and quality of life using validated instruments. Associations between disease severity, symptom burden, psychological measures, and quality of life were analyzed.

This was a cross-sectional observational study conducted among patients with HS. The diagnosis of HS was based on clinical examination by experienced dermatologists, in accordance with established diagnostic criteria [1]. Consecutive patients were recruited during routine dermatological consultations at tertiary referral centers in two cities in Poland: Wroclaw and Cracow. Demographic data collected included age, sex and disease duration. Clinical variables were asssessed by dermatologists and included disease severity. Subjective symptoms, such as pain and itch intensity, were scored by patients. Patients were also asked to fill out questionnaires related to psychological status (depression and anxiety) as well as QoL. All questionnaires were completed independently by the participants during the study visit, with assisstance available if clarification was needed. The study was carried out between June and November 2025. The study design is presented in Figure 1.

The study included 90 consecutive patients suffering from HS. 84 of them agreed to participate in the study, filled out all the necessary questionnaires and were considered for final analysis. This gave us a response rate of 93.3%. The study population included predominantly outpatients, as well as a small number of hospitalized patients (n = 2). In the Polish healthcare system, hospitalization of patients with HS is not exclusively associated with very severe disease and may be related to treatment qualification or diagnostic procedures. Importantly, disease severity of hospitalized patients did not differ from that observed in the outpatient group. The diagnosis of HS was based on clinical examination. Among the studied group, there were 43 females (51.2%) and 41 males (48.8%). Their mean age was 36.4 ± 12.3 years and the mean disease duration after the correct diagnosis was 5.7 ± 5.1 years (Table 1).

The study was conducted according the principles of Declaration of Helsinki. It was approved by local ethics committee (Ethics Committee of Lower Silesian Medical Chamber, Wroclaw, Poland—decision number 12/BNBP/2025); all included subjects gave informed consent prior to their participation in the current project.

### 2.2. Hidradenitis Suppurativa Clinical Severity Assessment

The severity of HS was evaluated using the Hurley staging system and the International Hidradenitis Suppurativa Severity Score System (IHS4) [16,17].

The Hurley staging system classifies patients into three categories based on the extent of lesions, the presence of scarring, and the formation of sinus tracts. Stage I is characterized by single or multiple inflammatory nodules or abscesses without scarring or sinus tracts. Stage II involves recurrent abscesses or nodules accompanied by sinus tract formation and scarring, often with several distinct lesions present. Stage III represents the most advanced form of the disease, with widespread involvement including multiple interconnected sinus tracts, abscesses, and extensive scarring [16].

The IHS4 provides a quantitative assessment of disease severity based on the number of nodules, abscesses, and draining tunnels. The score is calculated using the formula: (number of nodules × 1) + (number of abscesses × 2) + (number of draining tunnels × 4). According to the total score, HS severity is categorized as mild (up to 3 points), moderate (4–10 points), or severe (greater than 10 points) [17].

### 2.3. Pain and Itch Assessment

The intensity of pain and itch was evaluated using the Numeric Rating Scale (NRS). Participants were asked to indicate the severity of the most intense pain and itch they had experienced during the three days preceding the study, as well as the highest levels since the onset of their condition. Usually, the shorter recall period reduces the possible bias in the obtained results. The NRS is a unidimensional instrument commonly used to quantify the severity of symptoms such as pain and itch. It ranges from 0 (no itch/pain) to 10 (the worst imaginable pain/itch). For pain intensity, the NRS was categorized as ≤5—mild pain, 5–7—moderate pain, and 7–10—severe pain [18]. The Itch-NRS scores were interpreted as follows: 0—no itch, 1–3—mild itch, 4–6—moderate itch, 7–8—severe itch, and ≥9—very severe itch [19,20].

### 2.4. Depression and Anxiety Assessment

To assess depression and anxiety in the study population, several validated Polish-language self-administered questionnaires were used: the Hospital Anxiety and Depression Scale—Modified version (HADS-M) [21,22], the Patient Health Questionnaire-9 (PHQ-9) [23,24], and the Generalized Anxiety Disorder 7-item scale (GAD-7) [25,26].

The HADS-M is a modified version of the Hospital Anxiety and Depression Scale developed by Zigmond et al. [21]. It includes 16 items, each scored from 0 to 3 points. Maximum possible scores are 21 for both depression and anxiety subscales, and 6 for the aggression subscale. A score equal or greater than 8 is indicative of probable or possible depressive disorder/anxiety disorder [22].

The PHQ-9 consists of nine items corresponding to the major depressive disorder diagnostic criteria from the DSM-IV. Respondents rate how often they have experienced each symptom using a 4-point scale (0 = “not at all,” 1 = “several days,” 2 = “more than half the days,” 3 = “nearly every day”). Standard cutoff values are 5, 10, 15, and 20 points, indicating mild, moderate, moderately severe, and severe depression, respectively. A total score of ≥10 has a sensitivity and specificity of 88% for detecting major depressive disorder and is considered the diagnostic threshold [23,24].

The GAD-7 is a self-administered questionnaire that screens for generalized anxiety disorder and measures anxiety severity. It includes seven statements describing symptoms of tension and worry, each rated on a 4-point scale from 0 (“not at all”) to 3 (“nearly every day”). Higher scores indicate more severe anxiety. Cutoff values of 5, 10, and 15 correspond to mild, moderate, and severe anxiety, respectively, while a score of ≥8 is used as the threshold for probable generalized anxiety disorder [25,26].

It should also be emphasized that although established cut-off values of the PHQ-9 and GAD-7 indicate clinically relevant depressive or anxiety symptoms, a formal diagnosis of major depressive disorder or anxiety disorder requires a structured psychiatric interview conducted by a qualified mental health professional in accordance with DSM or ICD criteria.

The use of multiple instruments for the assessment of depression and anxiety was intentional and aimed to capture complementary dimensions of psychological distress. The PHQ-9 and GAD-7 are DSM-based tools that allow for a quantitative assessment of symptom severity, while the HADS-M was specifically designed for use in patients with somatic diseases and minimizes the influence of physical symptoms. This approach allowed for a more comprehensive evaluation of psychological burden in patients with HS.

### 2.5. Quality of Life (QoL) Assessment

Patients’ quality of life (QoL) was measured using the Polish version of the Dermatology Life Quality Index (DLQI) [27] and Hidradenitis Suppurativa Quality of Life (HiSQOL) questionnaires [28,29].

The DLQI is a dermatology-specific instrument designed to evaluate how skin diseases affect symptoms and feelings, daily activities, leisure, work and school performance, personal relationships, and treatment-related side effects over the preceding week. The tool consists of 10 questions, each scored from 0 to 3 points (0 = “not at all,” 1 = “a little,” 2 = “a lot,” 3 = “very much”) [27]. The total DLQI score, obtained by summing the responses, can range from 0 to 30 points. The interpretation is as follows: 0–1—no effect on QoL, 2–5—small effect, 6–10—moderate effect, 11–20—large effect, and 21–30—extremely large effect [28].

The HiSQOL is a 17-item questionnaire created in 2019 by Thorlacius et al. [29] through a collaborative effort between Danish and American specialists. It is designed to evaluate QoL of individuals with HS, based on experiences over the preceding seven days. Each question follows a Likert-type format, offering five or seven possible responses, with scores ranging from 0 (not at all) to 4 (extremely). Certain items also include options for cases where the activity is not applicable, either because it is irrelevant or cannot be performed due to HS severity. The HiSQOL covers various dimensions of HS impact, including physical symptoms, psychosocial and emotional effects, and lifestyle adaptations related to the disease [29,30].

The use of both a general dermatology-specific QoL instrument (DLQI) and an HS-specific questionnaire (HiSQOL) was intentional. While the DLQI allows comparison of QoL impairment across different dermatological conditions, the HiSQOL captures disease-specific aspects of HS that may not be adequately reflected by generic instruments. The combined use of these tools enables a more comprehensive assessment of both global and HS-specific QoL burden.

### 2.6. Statistical Analysis

All statistical analyses were conducted using IBM SPSS Statistics version 26 (SPSS Inc., Chicago, IL, USA). All analyses were performed on a final study population of 84 patients who completed all required assessments. Initially, data normality was verified using the Shapiro–Wilk test. Descriptive statistics including minimum, maximum, mean, standard deviation, median, and quartiles were calculated. Depending on the data distribution, comparisons of quantitative variables were performed using either Student’s *t*-test or the Mann–Whitney U test. Categorical variables were analyzed using the Chi-square test, for comparisons across more than two groups, ANOVA or the Kruskal–Wallis test was applied, followed by post hoc analyses with Bonferroni correction. A two-tailed *p*-value < 0.05 was considered statistically significant. Additionally, multiple linear regression analysis was performed to identify independent predictors of quality of life (QoL) impairment in patients with HS, with DLQI total score as the dependent variable. The model included age, sex, disease severity (IHS4), depressive symptoms (PHQ-9, HADS-D) and anxiety symptoms (GAD-7, HADS-A) entered simultaneously. Results were reported as unstandardized regression coefficients (B) with 95% confidence intervals and *p* values; model fit was summarized using R^2^ and adjusted R^2^.

## 3. Results

### 3.1. Clinical Severity of Hidradenitis Suppurativa; Presence and Intensity of Pain and Itch

Among the studied subjects, 13.1% of patients (n = 11) were diagnosed with Hurley stage 1, 71.4% (n = 60) with Hurley stage 2, and 15.5% (n = 13) with Hurley stage 3. The mean clinical severity according to the IHS4 was 9.2 ± 7.0 points, indicating moderate disease intensity. Mild HS was identified in 28.6% of patients (n = 24), 34.5% (n = 29) exhibited moderate HS, and 36.9% (n = 31) had severe disease (Table 1).

Cutaneous pain was reported by 95.2% of patients (n = 80) over the whole disease duration, with a mean severity score of 7.2 ± 2.1 points. During the last 3 days prior to the assessment, pain was present in 58.3% of patients (n = 49), with a mean intensity of 4.5 ± 2.4 points. Itch during the entire disease period was reported by 81.0% of patients (n = 68), and during the last 3 days by 53.6% (n = 45). The mean itch intensity was 5.5 ± 2.2 points for the whole disease duration and 3.5 ± 2.0 points for the last 3 days (Table 1).

### 3.2. Quality of Life

The mean DLQI score for the entire group was 9.9 ± 8.3 points, indicating a moderate impact on patients’ lives. Based on DLQI cut-off values, 9.5% of patients (n = 8) demonstrated an extremely large effect, 29.8% (n = 25) a very large effect, 29.8% (n = 25) a moderate effect, and 20.2% (n = 17) a small effect on quality of life. Only 10.7% of subjects (n = 9) reported normal quality of life. The mean HiSQOL score for all patients was 19.4 ± 15.2 points. The QoL scores did not significantly correlate (*p* > 0.05) with the disease duration.

### 3.3. Depression and Anxiety

**Figure 2:**
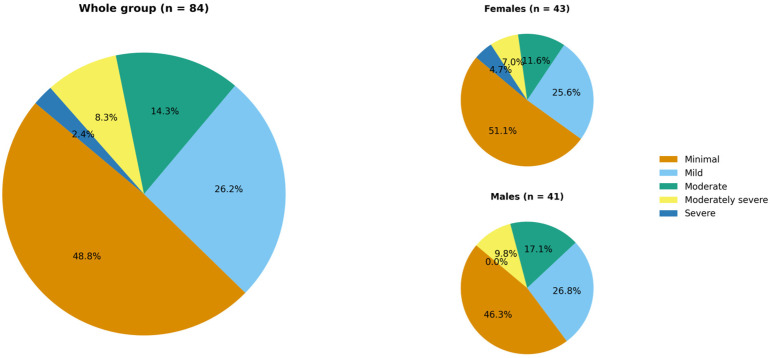
Hidradenitis suppurativa patients’ distribution across depression severity categories according to the PHQ-9 (no significant difference between females and males).

Based on the PHQ-9 questionnaire, 25.0% of patients (n = 21) met the criteria for probable or possible depressive disorder with a mean PHQ-9 score of 5.9 ± 5.7 points. Figure 2 shows the distribution of patients across depression severity categories according to the PHQ-9.

There was a statistically significant difference (*p* = 0.008) in PHQ-9 scores across Hurley stages. Post hoc analysis revealed that this difference (*p* = 0.06) was observed only between patients with Hurley stage 2 and stage 3 HS (4.9 ± 5.6 points and 9.5 ± 5.7 points, respectively). Similarly, HADS-D scores differed significantly (*p* = 0.026) across Hurley stages; post hoc analysis again confirmed a significant difference (*p* = 0.009) only between Hurley stage 2 and stage 3 patients (4.0 ± 3.6 points and 6.6 ± 3.2 points, respectively).

Moreover, PHQ-9 and HADS-D scores significantly correlated with IHS4 (r = 0.359, *p* < 0.001 and r = 0.346, *p* = 0.001, respectively). Regarding subjective symptoms, PHQ-9 scores positively correlated only with NRS itch during the last 3 days (r = 0.293, *p* = 0.048). In contrast, a weak, although significant (r = 0.216; *p* = 0.024), correlation was found between HADS-D scores and NRS pain intensity during the entire duration of the disease (Table 2).

**Figure 3:**
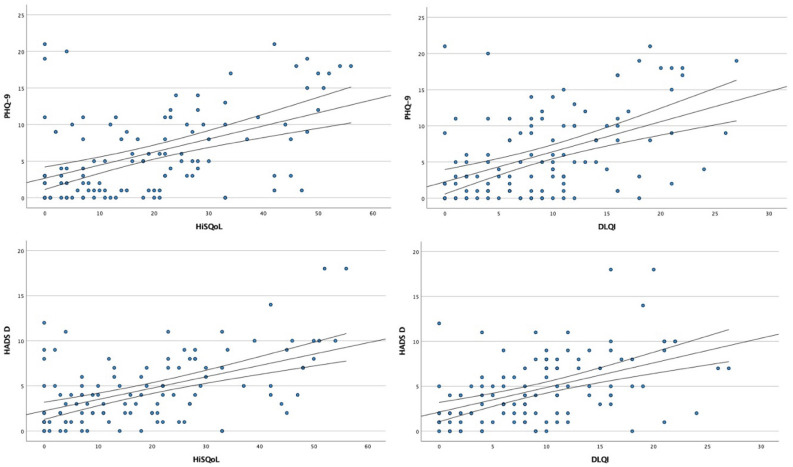
Scatter plots showing the correlation analysis between depressive symptoms and quality of life.

Additionally, we found a positive correlation between PHQ-9 scores and QoL assessments, both DLQI and HiSQOL (r = 0.530, *p* < 0.001 and r = 0.566, *p* < 0.001, respectively). HADS-D scores also correlated significantly with DLQI (r = 0.476, *p* < 0.001) and HiSQOL (r = 0.474, *p* < 0.001) (Figure 3).

**Figure 4:**
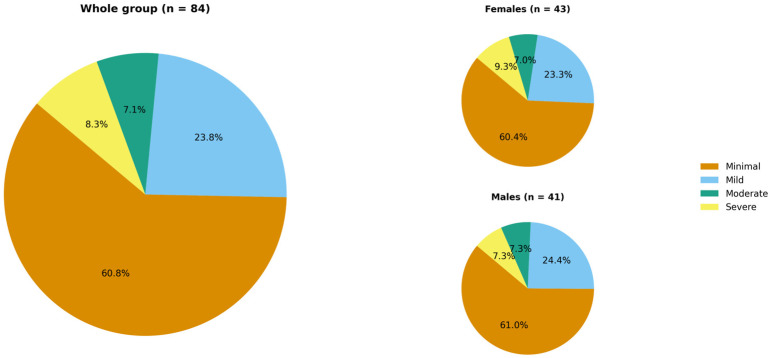
Hidradenitis suppurativa patients’ distribution across anxiety severity categories according to the GAD-7 (no significant difference between females and males).

In accordance with the GAD-7 questionnaire, a probable or possible anxiety disorder could be diagnosed in 15.5% of the study population (n = 13). The mean GAD-7 score was 4.8 ± 5.6 points. The distribution of patients across anxiety-severity categories is shown in Figure 4.

Neither GAD-7 nor HADS-A scores differed significantly among patients with different Hurley stages. However, both GAD-7 and HADS-A scores correlated positively with IHS4 scores (r = 0.220, *p* = 0.045 and r = 0.337, *p* = 0.002, respectively). GAD-7 scores correlated significantly with NRS pain assessments, both for the entire disease duration and for the last 3 days (r = 0.250, *p* = 0.026 and r = 0.307, *p* = 0.032, respectively). Interestingly, GAD-7 scores did not correlate with itch intensity, either for the whole disease duration or for the last 3 days. Although no relationship was observed between HADS-A scores and NRS pain scores for the entire disease period, a significant correlation was identified between HADS-A scores and pain assessments during the last 3 days (r = 0.402, *p* < 0.001). HADS-A scores also correlated with itch NRS scores from the last 3 days (r = 0.387, *p* = 0.002) (Table 3).

**Figure 5:**
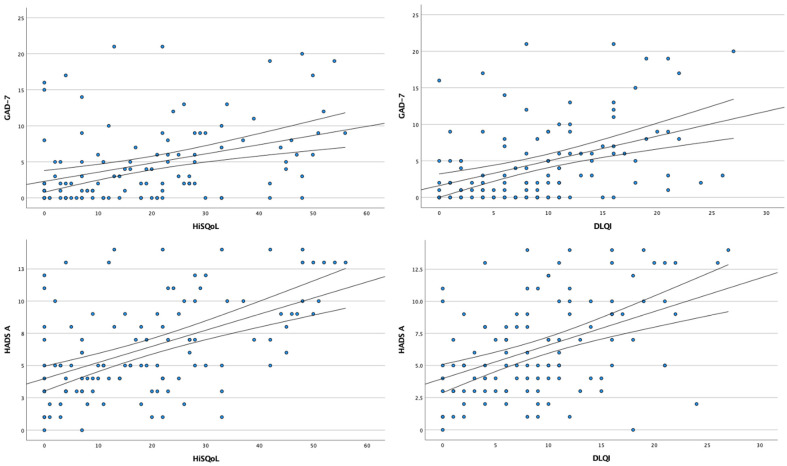
Scatter plots showing the correlation analysis between anxiety symptoms and quality of life.

Similar to PHQ-9, significant correlations were found between GAD-7 scores and DLQI (r = 0.472, *p* < 0.001) as well as HiSQOL (r = 0.468, *p* < 0.001). The same was observed for HADS-A scores, which correlated with both DLQI and HiSQOL (r = 0.437, *p* < 0.001 and r = 0.494, *p* < 0.001, respectively) (Figure 5). No statistically significant correlations (*p* > 0.05) were found between depression/anxiety questionnaire scores and disease duration.

### 3.4. Multivariable Linear Regression Analysis

In multivariable linear regression with DLQI as the dependent variable, higher HS severity (IHS4; B = 0.215, 95% CI 0.029–0.400, *p* = 0.024) and greater depressive symptom burden (PHQ-9; B = 0.342, 95% CI 0.32–0.651, *p* = 0.031) remained independent predictors of poorer QoL, whereas anxiety (GAD-7), HADS, age and sex were not significant contributors. The parameters of the model were as follows: R = 0.573, R^2^ = 0.328, Adjusted R^2^ = 0.284, *p* < 0.001.

To check if inclusion of hospitalized HS patients does not markedly influence the final results, we performed the sensitivity analysis. This analysis, excluding the two hospitalized patients, showed minimal changes in regression coefficients (IHS4: B = 0.215→0.190; PHQ-9: B = 0.342→0.362) with overlapping confidence intervals and preserved statistical significance for both predictors (*p* = 0.048 and *p* = 0.018, respectively), confirming the robustness of our findings.

## 4. Discussion

The present study demonstrates that both depression and anxiety are highly prevalent among individuals with HS, and that their intensity is closely related to clinical disease severity, subjective symptom burden, and QoL impairment. These findings reinforce the growing evidence that HS is not merely a dermatologic condition but a complex biopsychosocial disease with substantial psychiatric comorbidity. The proportion of patients presenting with clinically relevant depressive symptoms (25%) and anxiety symptoms (~15%) aligns with previous reports, although our prevalence estimates fall on the lower end of the spectrum compared with some studies [31,32]. Recent meta-analysis clearly showed that patients with HS are at increased risk for the development of depression (odds ratio: 2.25) and anxiety (odds ratio: 2.00) [33]. The observed differences in various studies may reflect variations in sample characteristics, assessment methods, or disease severity across cohorts.

A key finding of our study is the significant association between psychological symptoms and objective measures of HS severity. Both depressive and anxiety scores correlated with the IHS4, suggesting that more advanced disease is linked to greater psychiatric distress. Previous research has shown inconsistent relationships between psychiatric symptoms and HS severity; however, our results align with studies demonstrating higher depression and anxiety levels in patients with more severe clinical involvement [32,34]. This relationship likely reflects both the cumulative burden of symptoms such as pain, drainage, and itch, and the functional limitations imposed by severe disease. Notably, the correlations observed with pain and itch intensity are novel ones and further support the interconnectedness of somatic symptom burden and psychological distress. Pain and itch, two hallmark symptoms of HS, have previously been shown to independently impair QoL and contribute to sleep disturbance, perceived stigma, and emotional exhaustion [35,36,37,38]. The literature on the relationship between itch and depression is very scare [39,40]. Our findings extend this knowledge by demonstrating that both pain and itch in HS subjects may also be related to anxiety and, to some extent, depressive symptoms. It is worth mentioning that similar phenomena were reported in patients suffering from chronic hand eczema. Zalewski at al. [41]. showed that patients who scored higher on the PHQ-9 and HADS-D reported significantly greater severity of both pain and itch. In relation to possible anxiety disorders, they demonstrated a positive correlations between GAD-7 and HADS-A scorings and intensity of pain. Moreover, psoriasis patients with cutaneous pain scored higher on both depression and anxiety screening questionnaires [42].

QoL impairment emerged as a central component of the psychodermatological burden of HS. All psychological measures demonstrated strong correlations with both the DLQI and HiSQOL, emphasizing that emotional well-being is closely intertwined with daily functioning, social participation, and disease perception. This is consistent with prior qualitative and quantitative studies indicating that HS profoundly affects interpersonal relationships, sexual health, employment, and self-esteem [43,44]. Stigmatization, stemming from malodor, drainage, scarring, and visible lesions, remains a key driver of social withdrawal, shame, and isolation, which in turn exacerbate psychiatric vulnerability. The concept of cumulative life course impairment, recently proposed in relation to HS, is particularly relevant here: the psychological impact of the disease accumulates over time, shaping patients’ social trajectories, educational and occupational opportunities, and overall life satisfaction [45].

The interplay between HS and mental health is likely bidirectional. While the physical and psychosocial burden of HS contributes to the development of depressive and anxiety symptoms, growing evidence suggests potential shared biological mechanisms. Chronic systemic inflammation has been proposed as one of several potential contributors to psychiatric comorbidity in HS. Pro-inflammatory cytokines such as TNF-α, IL-17, and IL-6 have been variably associated with mood and anxiety disorders; however, published data remain inconsistent, with reports of both upregulation and downregulation depending on disease context, population, and methodology [1,46,47,48]. These observations suggest that inflammatory pathways may contribute to psychiatric vulnerability in HS in a complex and context-specific manner, rather than through a uniform mechanistic process. Such a situation may partially explain the high psychiatric vulnerability observed in HS populations and highlight the need for integrated therapeutic approaches that address both dermatologic and psychological domains.

In addition, anti-inflammatory treatments commonly used in HS and other inflammatory dermatological conditions may exert beneficial effects on depressive symptoms [49]. Targeted cytokine inhibitors have been shown to reduce depressive symptom severity, likely through attenuation of systemic inflammation and modulation of inflammatory pathways implicated in the pathophysiology of depression [49]. These observations further support the concept of a bidirectional relationship between inflammatory disease activity and mental health, whereby effective control of inflammation may lead not only to improvement of dermatological symptoms but also to better psychological outcomes.

The findings of this study may have potential clinical relevance; however, their reliance on self-reported measures indicates they should be interpreted with appropriate caution. First, our results underscore the necessity of routine mental health screening in HS care using brief, validated instruments such as the PHQ-9, GAD-7, and HADS. Despite strong evidence supporting the psychiatric burden of HS, standardized screening is still not routinely implemented in dermatology practice. Second, the correlations between subjective symptoms and psychological distress highlight the importance of adequate pain and itch management, both to improve physical comfort and to reduce the risk of psychiatric complications. Finally, our results support the broader push toward multidisciplinary care models integrating dermatologists, mental health professionals, pain specialists, and primary care providers. Such models can facilitate early detection, timely referrals, and targeted intervention, including psychotherapy, pharmacotherapy, and psychosocial support [1,44,45].

This study is not without limitations. Its cross-sectional design prevents causal inference, and reliance on self-report screening tools without confirmatory psychiatric evaluation may introduce diagnostic misclassification. Additionally, although our cohort reflects a real-world clinical population, the sample size is modest, and findings may not be fully generalizable to other regions or healthcare systems. A further limitation of this study is the potential construct overlap between disease-specific QoL measures and psychological symptom scales. The HiSQoL includes items partially related to emotional and social functioning, which may overlap with domains assessed by depression and anxiety questionnaires. As a result, the observed correlations may, to some extent, reflect shared underlying constructs rather than entirely independent associations. This overlap should be considered when interpreting the strength of these relationships. Future longitudinal studies are needed to explore the temporal dynamics between HS activity and mental health outcomes, evaluate the effects of treatment on psychological well-being, and identify predictive factors for psychiatric morbidity.

## 5. Conclusions

In summary, this study underscores the substantial psychological burden experienced by patients with HS. Depression and anxiety are common and closely linked to disease severity and QoL impairment. To the best of our knowledge, our study is the first to deeply analyze the relationship between psychiatric disturbances and subjective symptoms such as pain and itch. While our findings demonstrate significant associations between psychiatric symptoms, disease severity, and subjective symptom burden in HS, they should be considered with caution, underscoring the need for longitudinal studies to clarify causal relationships. In general, our findings highlight the necessity of a holistic, multidisciplinary approach to HS management that includes routine mental health assessment and targeted psychosocial support. Addressing the psychiatric dimension of HS may ultimately improve not only emotional well-being but also overall clinical outcomes.

## Figures and Tables

**Table 1:** Demographic and clinical characteristics of the study population (n = 84).

CHARACTERISTIC	Value
**NUMBER OF PATIENTS,** **n**	84
**SEX,** **n (%)**	
Female	43 (51.2%)
Male	41 (48.8%)
**AGE, YEARS**	36.4 ± 12.3
**DISEASE DURATION AFTER DIAGNOSIS, YEARS**	5.7 ± 5.1
**HURLEY STAGE,** **n (%)**	
Stage I	11 (13.1%)
Stage II	60 (71.4%)
Stage III	13 (15.5%)
**IHS4 SCORE**	9.2 ± 7.0
**HS SEVERITY ACCORDING TO IHS4,** **n (%)**	
Mild	24 (28.6%)
Moderate	29 (34.5%)
Severe	31 (36.9%)
**PAIN INTENSITY (NRS)**	
Whole disease duration	7.2 ± 2.1
Last 3 days	4.5 ± 2.4
**ITCH INTENSITY (NRS)**	
Whole disease duration	5.5 ± 2.2
Last 3 days	3.5 ± 2.0

Data are presented as mean ± standard deviation or number (percentage). HS—hidradenitis suppurativa; IHS4—International Hidradenitis Suppurativa Severity Score System; NRS—Numeric Rating Scale.

**Table 2:** Correlations between depressive scores and subjective symptoms assessment.

Symptom	Correlation	*p* Value
**PHQ-9**		
Pain (whole disease duration)	0.167	0.138
Pain (last 3 days)	0.185	0.203
Itch (whole disease duration)	0.042	0.734
Itch (last 3 days)	0.293	0.048
**HADS-D**		
Pain (whole disease duration)	0.216	0.024
Pain (last 3 days)	0.222	0.067
Itch (whole disease duration)	0.008	0.938
Itch (last 3 days)	0.155	0.225

PHQ-9—Patient Health Questionnaire-9; HADS-D—Hospital Anxiety and Depression Scale-Depression.

**Table 3:** Correlations between anxiety scores and subjective symptoms assessment.

Symptom	Correlation	*p* Value
**GAD-7**		
Pain (whole disease duration)	0.250	0.026
Pain (last 3 days)	0.307	0.032
Itch (whole disease duration)	0.066	0.595
Itch (last 3 days)	0.268	0.072
**HADS-A**		
Pain (whole disease duration)	0.094	0.329
Pain (last 3 days)	0.402	0.001
Itch (whole disease duration)	0.135	0.193
Itch (last 3 days)	0.387	0.002

GAD-7—Generalized Anxiety Disorder 7; HADS-A—Hospital Anxiety and Depression Scale-Anxiety.

## Data Availability

Data are available upon reasonable request from the corresponding authors.
